# Comparison of three malnutrition screening tools prior to allogeneic hematopoietic stem-cell transplantation

**DOI:** 10.3389/fnut.2023.1233074

**Published:** 2023-10-03

**Authors:** Reza Amiri Khosroshahi, Hamed Mohammadi, Maryam Barkhordar, Sheida Zeraattalab-Motlagh, Hossein Imani, Amirabbas Rashidi, Erfan Sadeghi, Simon Wilkins, Seyed Asadollah Mousavi

**Affiliations:** ^1^Department of Clinical Nutrition, School of Nutritional Sciences and Dietetics, Tehran University of Medical Sciences, Tehran, Iran; ^2^Hematology, Oncology and Stem Cell Transplantation Research Center, Tehran University of Medical Sciences, Tehran, Iran; ^3^Cell Therapy and Hematopoietic Stem Cell Transplantation Research Center, Tehran University of Medical Sciences, Tehran, Iran; ^4^Department of Community Nutrition, School of Nutritional Sciences and Dietetics, Tehran University of Medical Sciences (TUMS), Tehran, Iran; ^5^Department of Biostatistics, School of Medicine, Shiraz University of Medical Sciences, Shiraz, Iran; ^6^Cabrini Monash Department of Surgery, Cabrini Hospital, Melbourne, VIC, Australia; ^7^Department of Biochemistry and Molecular Biology, Monash University, Melbourne, VIC, Australia

**Keywords:** hematopoietic stem cell transplantation, leukemia, malnutrition, global leadership initiative on malnutrition, GLIM

## Abstract

**Background:**

Previous studies have shown that malnutrition before hematopoietic stem cell transplantation (HSCT) is associated with poor patient prognoses. There is inconsistency among studies on which nutritional status screening tool is appropriate for malnutrition diagnosis before allo-HSCT. The present study aimed to compare nutritional screening tools in patients with leukemia before allo-HSCT.

**Methods:**

An observational, cross-sectional, and single-center study was conducted in Tehran, Iran. One hundred four adults allo-HSCT candidates aged 18-55 years with leukemia were selected sequentially. Malnutrition assessment was done using three tools, the Global Leadership Initiative on Malnutrition (GLIM), nutritional risk screening 2002 (NRS-2002) and European Society for Clinical Nutrition and Metabolism (ESPEN) criteria. The agreement between malnutrition assessment tools was evaluated with Cohen’s kappa.

**Results:**

The agreement between GLIM and NRS-2002 was perfect (κ = 0.817, *p* < 0.001), while the agreement between GLIM and ESPEN was fair (κ = 0.362, *p* < 0.001). The agreement between NRS-2002 and ESPEN was fair (κ = 0.262, *p* < 0.001). We also found a moderate agreement for all tools (κ = 0.489, *p* < 0.001).

**Conclusion:**

NRS-2002 is an accepted tool for screening malnutrition in hospitalized patients. In the current study, the GLIM criterion perfectly agreed with the NRS-2002. Further studies in the HSCT setting are needed to introduce a valid tool.

## Introduction

Allogeneic hematopoietic stem cell transplantation (allo-HSCT) is a usual treatment for hematological diseases like leukemia (1). Even though life expectancy is incremented, treatment is related to a range of complications, among which changes in nutritional status (inadequate consumption or uptake of nutrients) have been shown as the most frequent (2, 3). Due to the disease, patients experience metabolic and endocrine alterations, which could provoke catabolic and inflammatory processes (4, 5). Furthermore, the nutritional status of patients may be affected due to gastrointestinal dysfunctions as a side effect of receiving various high-dose systematic radiation and/or chemical therapies before transplantation (6). Poor nutritional status increases the risk of emerging complications, including mucositis or fungal infections, higher mortality rate, and longer lengths of hospital stay (7, 8). Therefore, evaluating the nutritional status of HSCT patients with leukemia, providing nutritional support in advance, and maintaining patients in a well-nourished state are prominent for bone marrow and immune rebuilding (6, 9).

Few studies demonstrated nutritional screening tools to diagnose malnutrition in patients with hematological malignancies prior to HSCT, and none has presented conclusive evidence that verified which tool is obviously superior (10, 11). Among these tools, the European Society for Clinical Nutrition and Metabolism (ESPEN) (12) and nutritional risk screening 2002 (NRS-2002) criteria (13) have been widely used in oncology patients.

Notably, the Global Leadership Initiative on Malnutrition (GLIM) criteria were suggested by nutritional scientific societies in 2019 for diagnosing malnutrition (14). It consists of some phenotypic criteria (weight loss, decreases in body mass index (BMI), or loss of muscle reserve) and etiological criteria (decreased intake, acute or chronic stress) being prominent to recognize a minimum of one of each to be considered malnourished. Although GLIM criteria have been used in a few oncology studies, some showed that GLIM is sensitive and recommended for diagnosing malnutrition in hospitalized patients with cancer (15).

Until now, no study evaluates the malnutrition status of patients with leukemia who are candidates for allo-HSCT with GLIM criteria. A few studies compare the GLIM criteria with other previously utilized criteria, including NRS-2002 and ESPEN. We explained that NRS-2002 offers a holistic assessment, ESPEN’s evidence-based nature ensures a comprehensive framework, and GLIM criteria incorporate the latest advancements. This comparison enables us to identify the most appropriate criteria in the clinical setting. Therefore, this study aimed to compare the diagnostic test accuracy of the GLIM, NRS-2002, and ESPEN criteria for malnutrition diagnosis before allo-HSCT in patients with leukemia. This combination of tools ensures a thorough evaluation of nutritional risk in the context of allo-HSCT.

## Materials and methods

### Design and sample

An observational, cross-sectional, and single-center study was conducted in the Hematology Center of Shariati Hospital, Tehran, Iran. One hundred four adult allo-HSCT candidates aged 18–55 years with leukemia hospitalized in bone marrow transplant wards from November 2021 to December 2022 were selected sequentially. Patients with leukemia were eligible for inclusion if they were aged between 18 and 55 and candidates for allo-HSCT.

The exclusion criteria were refusal to be involved in this study, and patients who were candidates for autologous transplantation.

### Demographic and clinical assessments

Demographic variables such as age, sex (male or female), and clinical variables of the patients, which included the type of malignancy (acute myeloid leukemia (AML) or acute lymphoblastic leukemia (ALL)), complete remission (CR) status (CR = 1, CR = 2, CR = 3), and risk status (favorable, intermediate, and adverse) were extracted from the patient’s medical records.

### Functional status assessment

The functional status of patients was assessed based on the Karnofsky functional status index, which includes a score from 0 (dead) to 100 (normal) with 10-point intervals (16). A score of 80 (the patient can perform normal activities with effort) was considered the cut-off, and the patients were subdivided into two groups, >80 and ≤ 80.

### Anthropometric and body composition assessment

Body weight was measured using a digital scale (Seca, Hamburg, Germany) with an accuracy of 0.1 kg. Height was assessed using a tape measure attached to the wall and an accuracy of 0.5 cm. BMI was calculated by dividing weight (kg) by the square of height (m^2^).

The Tanita (BC-418’s) device was used to evaluate body composition during fasting conditions, with minimal water consumption and little exercise before the test and after defecation. This device offers independent analysis for various organs (right and left hand and right and left foot) and the trunk due to employing eight electrodes (two beneath the right foot and two in the right and left hand). Before the assessment, all the anthropometric devices were calibrated.

### Dietary assessment

Daily calorie intake and consumption of macronutrients (carbohydrate, protein, and fat) of all patients was checked by a trained dietitian with 3-day 24-h dietary recalls (2 non-consecutive normal days and 1 day off). The recorded amount of each food using the scale guide for Iranian households was converted to grams (17), and analyzed using the United States Department of Agriculture food composition database (18).

### Malnutrition assessment

Nutritional assessment was conducted by a trained evaluator who remained blinded during the initial 48 h of hospitalization. Three tools were utilized for this purpose: the NRS-2002 for nutritional screening to identify patients at risk, and the GLIM and ESPEN criteria for diagnosing malnutrition. The GLIM criteria requires at least one phenotypic and etiological criteria for malnutrition diagnosis (14). Phenotypic criteria include unwanted weight loss (weight loss of 5–10% in 6 months or 10–20% in more than 6 months), age-related BMI (>20 kg/m^2^ for <70 years and < 22 kg/m^2^ for ≥70 years) and reduced muscle mass. In the current study, the reduction of muscle mass was considered based on Appendicular Skeletal Muscle Index (ASMI, kg/m2) <7 for men and ASMI <5.7 for women. Etiological criteria include reduced energy expenditure, chronic gastrointestinal status, disease burden, and inflammatory conditions. According to the definition of GLIM criteria, disease burden, and inflammation were considered positive in all patients in the current study.

Assessment of nutritional status based on NRS-2002 is done using a questionnaire. The main components of this questionnaire were: 1) the severity of the impact of the primary disease on the nutritional status; 2) recent changes in body weight (during the last one to 3 months); 3) Change in food intake during the last week; 4) BMI; If the evaluated person is 70 years old or more, one point will be added to the sum of her points. NRS score equal to 3 and more than 3 is defined as a nutritional risk (13).

The ESPEN criteria have proposed two methods to diagnose malnutrition. The first method is BMI <18.5, and the second method is a combination of unwanted weight loss (more than 10% of normal weight in an unlimited time or more than 5% in 3 months) along with low BMI (<22 kg/m^2^ for patients ≥70 years old and < 20 kg/m^2^ for <70 years) or low fat-free mass index (FFMI) (<15 kg/m^2^ in women and < 17 kg/m^2^ in men) (19).

### Calculation of sample size

According to a previous study comparing NRS2002 and GLIM (20) with area under the curve (AUC) of 0.896, considering alpha = 0.05, power = 80%, and precision of 10%, the minimum required sample size was estimated to be 94 patients using PASS 2023 Power Analysis and Sample Size Software (2023). NCSS, LLC. Kaysville, Utah, United States, ncss.com/software/pass. Therefore, taking into account 10% drop-out rate, the sample size was finally determined to be 104 patients (21).

### Statistical analysis

The Kolmogorov–Smirnov test was used to evaluate the normality of continuous variables. Demographic, clinical, and anthropometric variables, as well as dietary intakes of patients in accordance with malnutrition status (defined by GLIM, NRS-2002, and ESPEN), were evaluated by independent sample t-test or Mann–Whitney test and Chi-squared or Fisher’s exact test for continuous and categorical variables, respectively. The agreement between the two malnutrition assessment tools was evaluated with Cohen’s kappa (22). Fleiss’ kappa was used to assess the agreement between three malnutrition screening tools (23). The agreement of the instruments in subgroups, including sex, type of leukemia, CR, and risk status, were also examined. The Kappa value varies from 0 to 1, and its interpretation is as follows: <0.2 is weak, 0.2–0.4 is fair, 0.4–0.6 is moderate, 0.6–0.8 is substantial, and > 0.8 is perfect. Sensitivity, specificity, positive predictive value (PPV), and negative predictive value (NPV) were calculated to assess the effectiveness of diagnostic tests. *p* value <0.05 was considered significant. Statistical analysis was performed with SPSS (version 24.0 Armonk, New York, NY, United States).

### Ethical considerations

The ethics committee of Tehran University of Medical Sciences evaluated and approved this project (Ethics code: IR.TUMS.MEDICINE.REC.1400.1089). A signed consent form was obtained from all the participants.

## Results

Table 1 presents demographic and clinical variables according to the GLIM, NRS-2002, and ESPEN criteria. In total, 104 patients (41.3% female) were recruited. The mean ± SD age of the patients was 35.5 ± 10.4 years (median = 38 years, range = 19–55). AML was the most frequent type of leukemia (*n* = 67, 64.4%). The GLIM and NRS-2002 indicated the highest percentage of patients with malnutrition (26 and 33.6%), and the ESPEN criteria indicated the lowest percentage of patients with malnutrition (8.65%). Patients without malnutrition were more prone to be in the first CR for all tools (all *p* < 0.05). The NRS-2002 showed that patients without malnutrition were more prone to AML than ALL (*p* = 0.049); however, the adverse risk of disease was higher (*p* = 0.012).

**Table 1:** Demographic and clinical variables according to the GLIM criteria, NRS-2002, and ESPEN criteria.

Variables	Total (*n* = 104)	GLIM criteria		*p*-value	NRS-2002		*p*-value	ESPEN criteria		*p*-value
		No malnutrition (*n* = 77)	Malnutrition (*n* = 27)		No malnutrition (*n* = 69)	Malnutrition (*n* = 35)		No malnutrition (*n* = 95)	Malnutrition (*n* = 9)	
*Gender, n (%)*										
Male	61 (58.7)	46 (59.7)	15 (55.6)	0.704	44 (63.8)	17 (48.6)	0.137	37 (38.9)	6 (66.7)	0.157^a^
Female	43 (41.3)	31 (40.3)	12 (44.4)		25 (36.2)	18 (51.4)		58 (61.1)	3 (33.3)	
*Age (years)*	35.50 ± 10.37	38.61 ± 10.75	38.19 ± 38	0.856	39.03 ± 10.87	37.46 ± 9.38	0.468	38.95 ± 10.56	33.78 ± 6.83	0.154
*Type of leukemia, n (%)*										
AML	67 (64.4)	53 (68.8)	14 (51.9)	0.113	49 (71.0)	18 (51.4)	**0.049**	64 (67,4)	3 (33.3)	0.065^a^
ALL	37 (35.6)	24 (31.2)	13 (48.1)		20 (29.0)	17 (48.6)		31 (32.6)	6 (66.7%)	
*CR, n (%)*										
CR = 1	79 (76.0)	63 (81.8)	16 (59.3)	**0.008**	58 (84.1)	21 (60.0)	**0.011**	73 (76.8)	6 (66.7)	**<0.001**
CR = 2	20 (19.2)	13 (16.9)	7 (25.9)		10 (14.5)	10 (28.6)		20 (21.1)	0 (0)	
CR = 3	5 (4.8)	1 (1.3)	4 (14.8)		1 (1.4)	4 (11.4)		2 (2.1)	3 (33.3)	
*Risk status, n (%)*										
Favorable	3 (2.9)	2 (3.1)	1 (3.7)	0.054	2 (3.6)	1 (2.9)	**0.012**	2 (2.4)	1 (11.1)	0.202
Intermediate	12 (11.5)	12 (18.8)	0 (0)		12 (21.4)	0 (0)		12 (14.6)	0 (0)	
Adverse	76 (83.5)	50 (78.1)	26 (96.3)		42 (75)	34 (97.1)		68 (82.9)	8 (88.9)	
*Functional status, n (%)*										
>80	101 (97.1)	76 (98.7)	25 (92.6)	0.164^a^	68 (98.6)	33 (94.3)	0.26^a^	94 (98.9)	7 (77.8)	**0.019** ^**a**^
≤80	3 (2.9)	1 (1.3)	2 (7.4)		1 (1.4)	2 (5.7)		1 (1.1)	2 (22.2)	

AML, Acute myeloid leukemia; ALL, Acute lymphocytic leukemia; CR, complete remission; GLIM, Global Leadership Initiative on Malnutrition; NRS-2002, Nutrition Risk Screening 2002.

The data are presented as mean ± standard deviation (SD) or percent.

a Calculated by Fisher test. We meant to use bold to show statistical significance (*p* < 0.05).

Anthropometric variables of patients according to the GLIM, NRS-2002, and ESPEN criteria are summarized in Table 2. According to three screening tools, patients not suffering from malnutrition were more prone to having higher weight, BMI, FFMI, and ASM than those with malnutrition (all *p* < 0.05). Although GLIM and ESPEN criteria indicated that FM was significantly higher in patients without malnutrition, this relation was insignificant based on NRN-2002. No significant difference was observed between malnourished and well-nourished groups for all tools regarding BF (all *p* > 0.05).

**Table 2:** Anthropometric variables according to the GLIM criteria, NRS-2002, and ESPEN criteria.

Variable	Total (*n* = 104)	GLIM criteria		*p*-value	NRS-2002		*p*-value	ESPEN criteria		*p*-value
		No malnutrition (*n* = 77)	Malnutrition (*n* = 27)		No malnutrition (*n* = 69)	Malnutrition (*n* = 35)		No malnutrition (*n* = 95)	Malnutrition (*n* = 9)	
Anthropometric variables										
Weight (kg)	78.87 ± 15.49	81.95 ± 14.33	70.11 ± 15.58	**<0.001**	81.88 ± 14.79	72.96 ± 15.37	**0.005**	81.10 ± 14.10	55.40 ± 8.68	**<0.001**
BMI (kg/m^2^)	26.49 ± 4.35	27.51 ± 3.61	23.58 ± 5.01	**<0.001**	27.37 ± 3.61	24.76 ± 5.17	**0.010**	27.19 ± 3.81	19.10 ± 2.45	**<0.001**
FFM (kg)	57.97 ± 13.11	59.53 ± 12.87	53.51 ± 13.01	**0.043** ^**a**^	60.29 ± 12.86	53.39 ± 12.56	**0.012** ^**a**^	59.34 ± 12.58	43.41 ± 9.62	**0.001** ^**a**^
FM (kg)	20.91 ± 8.98	22.41 ± 8.37	16.61 ± 9.44	**0.003** ^**a**^	21.59 ± 8.21	19.56 ± 10.35	0.294 ^a^	21.75 ± 8.72	11.98 ± 6.95	**0.003** ^**a**^
BF (%)	26.33 ± 9.64	27.40 ± 9.04	23.29 ± 10.79	0.056	26.39 ± 8.59	26.22 ± 11.58	0.942	26.78 ± 9.41	21.57 ± 11.36	0.122
ASM (kg)	25.84 ± 6.38	26.65 ± 6.27	23.52 ± 6.24	**0.027** ^**a**^	27.01 ± 6.22	23.53 ± 6.14	**0.008**	26.52 ± 6.12	18.62 ± 4.52	**<0.001** ^**a**^

BF, body fat; BMI, body mass index; FFM, fat free mass; FM, fat mass; ASM, Appendicular Skeletal Muscle; GLIM, Global Leadership Initiative on Malnutrition; NRS-2002, Nutrition Risk Screening 2002; SMM, skeletal muscle mass.

The data are presented as mean ± standard deviation (SD).

a Calculated by Mann–Whitney test. We meant to use bold to show statistical significance (*p* < 0.05).

The detailed results of the sensitivity, specificity, positive and negative predictive values for all patients and according to sex, type of leukemia, CR, and risk of disease are available in Table 3. All findings were subdivided based on the two comparisons: 1) GLIM vs. NRS-2002, 2) GLIM vs. ESPEN, and 3) ESPEN vs. NRS-2002.

**Table 3:** Statistical results comparing GLIM criteria, NRS-2002, and ESPEN criteria in all patients and according to sex, type of leukemia, CR, and risk of disease.

Tools	Sensitivity % (95% CI)	Specificity % (95% CI)	Positive predictive value % (95% CI)	Negative predictive value % (95% CI)	kappa value (P)	Kappa Fleiss (P)^a^
**All patients**						0.489 (**<0.001**)
GLIM vs. NRS-2002	100 (94.79 to 100)	77.14 (59.86 to 89.58)	89.61 (82.43 to 94.07)	100 (NA)	0.817 (**<0.001**)	
GLIM vs. ESPEN	80 (70.54 to 87.51)	88.89 (51.75 to 99.72)	98.70 (92.27 to 99.79)	29.63 (20.94 to 40.10)	0.362 (**<0.001**)	
ESPEN vs. NRS-2002	93.55 (92.19 to 99.96)	22.86 (10.42 to 40.14)	71.58 (67.72 to 75.14)	88.89 (51.02 to 98.40)	0.262 (**<0.001**)	
**Sex**						
*Male*						0.505 (**<0.001**)
GLIM vs. NRS-2002	100 (91.96 to 100)	88.24 (63.56 to 98.54)	95.65 (85.68 to 98.78)	100 (NA)	0.915 (**<0.001**)	
GLIM vs. ESPEN	79.31 (66.65 to 88.83)	100 (29.24 to 100)	100	20 (13.12 to 29.27)	0.274 (**0.002**)	
ESPEN vs. NRS-2002	100 (91.96 to 100)	17.65 (3.80 to 43.43)	75.86 (71.61 to 79.66)	100 (NA)	0.236 (**0.004**)	
*Female*						0.461 (**<0.001**)
GLIM vs. NRS-2002	100 (86.28 to 100)	66.67 (40.99 to 100)	80.65 (68.43 to 88.90)	100 (NA)	0.699 (**<0.001**)	
GLIM vs. ESPEN	81.08 (64.84 to 92.04)	83.33 (35.88 to 99.58)	96.77 (83.27 to 99.45)	41.67 (25.10 to 60.36)	0.454 (**0.001**)	
ESPEN vs. NRS-2002	96 (79.65 to 99.90)	27.78 (9.69 to 53.48)	64.86 (57.83 to 71.31)	83.33 (38.93 to 97.51)	0.262 (**0.026**)	
**Type of leukemia**						
*AML*						0.400 (**0.008**)
GLIM vs. NRS-2002	100 (92.75 to 100)	77.78 (52.36 to 93.59)	92.45 (83.77 to 96.67)	100 (NA)	0.837 (**<0.001**)	
GLIM vs. ESPEN	81.25 (69.54 to 89.92)	66.67 (9.43 to 99.16)	98.11 (91.27 to 99.62)	14.29 (6.06 to 30.09)	0.174 (**0.046**)	
ESPEN vs. NRS-2002	97.96 (89.15 to 99.95)	11.11 (1.38 to 34.71)	75.0 (71.71 to 78.02)	66.67 (16.17 to 95.40)	0.123 (0.112)	
*ALL*						0.4969 (<0.001)
GLIM vs. NRS-2002	100 (83.16 to 100)	76.47 (50.10 to 93.19)	83.33 (67.97 to 92.18)	100 (NA)	0.778 (<0.001)	
GLIM vs. ESPEN	77.42 (58.90 to 90.41)	100 (54.07 to 100)	100 (NA)	46.15 (30.87 to 62.19)	0.527 (<0.001)	
ESPEN vs. NRS-2002	100 (83.16 to 100)	35.29 (14.21 to 61.67)	64.52 (56.14 to 72.09)	100 (NA)	0.371 (0.004)	
**CR**						
*CR = 1*						0.517 (**<0.001**)
GLIM vs. NRS-2002	100 (93.84 to 100)	76.19 (52.83 to 91.78)	92.06 (84.37 to 96.14)	100 (NA)	0.825 (**<0.001**)	
GLIM vs. ESPEN	84.93 (74.64 to 92.23)	83.33 (35.88 to 99.58)	98.41 (91.18 to 99.73)	31.25 (19,15 to 46.59)	0.387 (**<0.001**)	
ESPEN vs. NRS-2002	98.28 (90.76 to 99.96)	23.81 (8.22 to 47.17)	78.08 (73.67 to 81.94)	83.33 (38.25 to 97.58)	0.286 (**0.001**)	
*CR = 2*						0.179 (0.432)
GLIM vs. NRS-2002	100 (69.15 to 100)	70.0 (34.75 to 93.33)	76.92 (56.39 to 89.57)	100 (NA)	0.700 (**0.001**)	
GLIM vs. ESPEN	–	–	–	–	–	
ESPEN vs. NRS-2002	–	–	–	–	–	
*CR = 3*						0.659 (**0.011**)
GLIM vs. NRS-2002	100 (2.5 to 100)	100 (39.76 to 100)	100 (NA)	100 (NA)	1.000 (**0.025**)	
GLIM vs. ESPEN	50 (1.26 to 98.74)	100 (29.24 to 100)	100 (NA)	75.0 (42.87 to 92.31)	0.545 (0.171)	
ESPEN vs. NRS-2002	100 (2.50 to 100)	75.0 (19.41 to 99.37)	50.0 (15.48 to 84.52)	100 (NA)	0.545 (0.171)	
**Risk status**						
*Favorable*						1.000 (**0.003**)
GLIM vs. NRS-2002	100 (15.18 to 100)	100 (2.50 to 100)	100 (NA)	100 (NA)	1.000 (0.083)	
GLIM vs. ESPEN	100 (15.81 to 100)	100 (2.50 to 100)	100 (NA)	100 (NA)	1.000 (0.083)	
ESPEN vs. NRS-2002	100 (15.81 to 100)	100 (2.50 to 100)	100 (NA)	100 (NA)	1.000 (0.083)	
**Adverse**						0.413 (**<0.001**)
GLIM vs. NRS-2002	100 (91.59 to 100)	76.47 (58.83 to 89.25)	84.0 (74.12 to 90.59)	100 (NA)	0.782 (**<0.001**)	
GLIM vs. ESPEN	72.06 (59.85 to 82.27)	87.50 (47.35 to 99.68)	98.0 (88.62 to 99.68)	26.92 (18.82 to 36.92)	0.299 (**0.001**)	
ESPEN vs. NRS-2002	97.92 (88.93 to 99.95)	20.59 (8.70 to 37.90)	63.51 (59.35 to 67.49)	87.50 (47.43 to 98.19)	0.196 (**0.010**)	

AML, Acute myeloid leukemia; ALL, Acute lymphocytic leukemia; CR, complete remission; CI, confidence interval; NA, not applicable.

a Indicating the agreement between the three instruments. We meant to use bold to show statistical significance (*p* < 0.05).

### Results for all patients

GLIM indicated sensitivity and specificity of 100 and 77.14% when comparing the GLIM with NRS-2002 as a reference tool, and 80 and 88.89% when comparing the GLIM with ESPEN as reference tool, the agreement between GLIM and NRS-2002 was perfect (κ = 0.817, *p* < 0.001), while the agreement between GLIM and ESPEN was fair (κ = 0.362, *p* < 0.001). Moreover, when comparing the ESPEN with NRS-2002 as a reference tool, ESPEN showed a sensitivity of 93.55%, while the specificity was 22.86%. The agreement between these two instruments was fair (κ = 0.262, *p* < 0.001). We also found a moderate agreement between GLIM, NRS-2002, and ESPEN (κ = 0.489, *p* < 0.001) (Table 3).

### Results per gender

In male patients, the agreement between GLIM and NRS-2002 was perfect (κ = 0.915, *p* < 0.001); however, the agreement between GLIM and ESPEN (κ = 0.274, *p* = 0.002), and between ESPEN and NRS-2002 (κ = 0.236, *p* = 0.004) were fair. We also found a moderate agreement between GLIM, NRS-2002, and ESPEN (κ = 0.505, *p* < 0.001). In female patients, the agreement between GLIM and NRS-2002 was substantial (κ = 0.699, *p* < 0.001), and the agreement between GLIM and ESPEN was moderate (κ = 454, *p* = 0.001). However, the agreement between ESPEN and NRS-2002 was fair (κ = 0.262, *p* = 0.026). We also found a moderate agreement between GLIM, NRS-2002, and ESPEN (κ = 0.461, *p* < 0.001) (Table 3).

### Results per type of leukemia

In AML patients, the agreement between GLIM and NRS-2002 was perfect (κ = 0.837, *p* < 0.001), while the agreement between GLIM and ESPEN was slight (κ = 0.174, *p* = 0.046). There was no significant agreement between ESPEN and NRS-2002. We observed a fair deal between these three tools (κ = 0.400, *p* = 0.008). In ALL patients, our results indicated substantial agreement between GLIM and NRS-2002 (κ = 0.778, *p* < 0.001) and moderate agreement between GLIM and ESPEN (κ = 0.527, p < 0.001). Nevertheless, there was a fair agreement between ESPEN and NRS-2002 (κ = 0.371, *p* = 0.004). Also, the agreement between these three tools was moderate (κ = 0.496, *p* < 0.001) (Table 3).

### Results per complete remission

In patients in the first CR, the agreement between GLIM and NRS-2002 was perfect (κ = 0.825, *p* < 0.001), while the agreement between GLIM and ESPEN (κ = 0.387, *p* < 0.001) and between ESPEN and NRS-2002 (κ = 0.286, *p* = 0.001) were fair. We found a moderate agreement between all three mentioned tools (κ = 0.517, *p* < 0.001). In patients who achieved a second CR, the agreement between GLIM and NRS-2002 was substantial (κ = 0.700, *p* = 0.001). The agreement between GLIM, ESPEN, and NRS-2002 was not significant. In patients in the third CR, the agreement between GLIM and NRS-2002 was perfect (κ = 1.000, *p* = 0.025), while the agreement between GLIM and ESPEN and between ESPEN and NRS-2002 were insignificant. We found a substantial agreement between these three tools (κ = 0.659, *p* = 0.011) (Table 3).

### Results per risk status

In patients with a favorable risk of disease, although we observed no significant agreement between tools when comparing them two by two, a perfect agreement between GLIM, ESPEN, and NRS-2002 was seen (κ = 1.000, *p* = 0.003). In patients with adverse risk of disease, the agreement between GLIM and NRS-2002 was substantial (κ = 0.782, *p* < 0.001); however, the agreement between GLIM and ESPEN (κ = 0.299, *p* = 0.001) and ESPEN and NRS-2002 (κ = 0.196, *p* = 0.010) were fair and slight, respectively. We also found a moderate agreement between these three tools (κ = 0.413, *p* < 0.001) (Table 3).

## Discussion

This study is one of the first to compare different tools for evaluating malnutrition in patients with leukemia who are also candidates for allo-HSCT. According to NRS-2002, GLIM, and ESPEN criteria, the results showed that 33.6, 26, and 8.6% of the patients were malnourished, respectively. Regardless of gender, type of leukemia, CR status, and risk status, GLIM and NRS-2002 instruments had a perfect agreement in diagnosing malnutrition; however, the ESPEN criterion showed fair agreement with the other two instruments (GLIM and NRS-2002). In all patients, we obtained a moderate agreement for all tools. In general, these results were maintained in subgroup analyses.

Although malnutrition caused by disease is known as a serious problem, a gold standard for its diagnosis has not been introduced so far. While Peng et al. introduced the NRS-2002 as the first choice for assessing malnutrition before HSCT (24), studies have shown contradictions (10, 25). In 2017, the ESPEN guideline published a lack of consensus on the appropriate malnutrition screening method in patients with cancer (12). The GLIM criterion was developed in 2019 and now has a global consensus and has been evaluated in various diseases in recent years (14).

Most previous studies evaluated malnutrition in patients with solid tumors, and studies in HSCT candidate patients are limited. However, the nutritional status of patients with hematologic malignancies is not vastly different from general oncology patients (26). Various screening tools have led to different results in the prevalence of malnutrition in patients with hematological malignancies. For example, the prevalence of malnutrition using Subjective Global Assessment (SGA), Malnutrition Screening Tool (MST), Malnutrition Universal Screening Tool (MUST), Mini Nutritional Assessment (MNA), Patient-Generated Subjective Global Assessment (PG-SGA), and BMI tools varied from 13 to 41.3% (27–29).

The prevalence of malnutrition in this study based on the GLIM criteria was 26%, which was in line with previous studies in cancer patients. Previous studies have reported that the rate of malnutrition was 32% in head and neck cancer (30), 24% in lung cancer (31), and 72.2–80% in advanced stages of cancer based on GLIM criteria (32). In studies that included all types of cancer with larger sample sizes (637 and 2,794 patients), malnutrition was between 26 and 31% (20, 33). The current study’s results were consistent with previous studies in patients with hematological malignancies. In a study that examined 120 leukemia, lymphoma, and myeloma patients, the prevalence of malnutrition based on the GLIM criteria was 25.8%, which was 29.7% in leukemia patients (34). In lymphoma and leukemia patients, the prevalence of malnutrition significantly differs between different types of blood malignancies (28, 35). In China, Guo et al. (36) evaluated the prevalence of malnutrition based on the GLIM criteria in 98 HSCT candidate patients, which was 80.6%. One of the possible reasons for the difference in the results of the current study with the results of Guo et al.’s (35) study was the difference in the ethnicity of the study cohort. Guo et al. considered the difference in muscle tissue in the Chinese population with other races as the reason for this difference. They proposed the GILM criterion without reduced muscle tissue for the Chinese people.

In this study, our choice of comparing these tools stemmed from a prior investigation that pitted the NRS-2002 against the SGA. The findings revealed a high degree of concordance, with NRS-2002 exhibiting a specificity of 94.81% in identifying severe malnutrition. This strong agreement might be attributed to NRS-2002’s balanced consideration of both nutritional status and disease severity. It implies that NRS-2002 could categorize patients as high-risk primarily due to the severity of their illness (37).

Hence, the primary objective of our study was to assess NRS-2002 against alternative tools like the GLIM criteria, focusing on their specificity and sensitivity. While the PG-SGA is widely regarded as the gold standard for cancer patients, our study specifically aimed to compare screening and assessment tools commonly used in the pre-transplant setting. The choice of which method to employ depends on factors such as available infrastructure, resources, automation feasibility, and the healthcare environment (38).

Moreover, previous research has indicated that GLIM criteria demonstrate ‘moderate agreement’ (kappa = 0.426) when compared to SGA. This suggests that GLIM criteria can effectively assess the nutritional status of cancer patients even without considering SGA, a recommendation supported by existing literature (15, 39). Additionally, large-scale prospective research by Tan et al. indicated that GLIM criteria demonstrated “moderate agreement” (kappa = 0.76) and good reliability with SGA. Prediction of nutritional and functional status, cancer-associated symptoms and quality of life can be done by applying GLIM (40). In addition, a cross-sectional study among colorectal cancer patients showed that malnutrition frequency according to GLIM criteria can be recorded with/without considering the screening tools (41). Another survey among esophageal cancer patients indicated that between GLIM, SGA, and ESPEN criteria, the GLIM indicated a greater malnutrition prevalence rate and seemed to be the optimal framework for predicting post-surgery complications (42). We might conclude that the implication of criteria with higher sensitivity, including the GLIM criteria, might assist early diagnosis and, therefore, early intervention in patients with cancer.

The results of our study determined that the GLIM and ESPEN criteria have a fair agreement in diagnosing malnutrition in leukemia patients who are candidates for HSCT. In the subgroup analysis, the agreement ranged from weak to moderate; however, in the group of patients with favorable risk at the beginning of transplantation, a perfect agreement was shown, but because only three patients were in that group, the clinical significance of the result is reduced, and further studies are needed. The results of our study were consistent with previous studies; for example, Ruiz et al. determined the prevalence of malnutrition in outpatient cancer patients based on the GLIM criteria, 46.7%, and based on the ESPEN criteria, 17.6% (39). The kappa value between the two instruments was 0.34, which indicates a fair agreement. A fair agreement between the GLIM and the ESPEN criterion has been reported in studies with a population of various cancers and esophageal cancer [35; 44]. In general, the amount of malnutrition based on the ESPEN criteria is estimated to be lower than the GLIM criteria.

One of the important reasons for the difference in the prevalence of malnutrition obtained with the above two tools is the reduction of the BMI cut-off required to diagnose malnutrition in the ESPEN criteria. In addition to all the parameters for diagnosing malnutrition based on ESPEN, the GLIM criterion also has a series of etiological criteria that can be effective in the high prevalence of malnutrition compared to the ESPEN criterion.

The NRS-2002 and GLIM criteria perfectly agreed in diagnosing malnutrition in this study. In the subgroup analysis, the agreement ranged from substantial to perfect. 33.6% of patients in the current research based on NRS-2002 were recognized as being at nutritional risk, comparable to previous studies in patients with cancer and candidates for HSCT (10, 20). Among 637 patients with cancer, the GLIM criterion had the best agreement with the NRS-2002 instrument compared to several other questionnaires (20). The higher agreement between NRS-2002 and GLIM criteria in this study and previous studies is most likely due to the similarity of NRS-2002 measures (weight, BMI, food intake, and disease severity) to determine malnutrition with GLIM criteria.

Malnutrition assessment is important because its early diagnosis leads to better and more effective interventions in the hospital. Early intervention prevents the progression of malnutrition to cancer cachexia (43). Various classifications have been proposed for cancer cachexia, but they all have the exact definition and introduce cachexia as an irreversible stage in that even the use of some nutritional supplements or drugs can be a moral conflict (44). Cachexia has three stages: pre-cachexia, cachexia, and refractory cachexia. The stage of pre-cachexia refers to weight loss below 5%; Cachexia includes BMI <20, weight loss of more than 5% in the last 6 months, or sarcopenia. BMI <20 is similar to the BMI cut-off in the ESPEN criteria. Therefore, the ESPEN criterion may have problems diagnosing patients in the stage of pre-cachexia. Diagnosing the stage of pre-cachexia leads to a better effect of nutritional treatment.

### Strengths and limitations

One of the strengths of this study was that the total population was patients with leukemia who were candidates for allo-HSCT, which had low heterogeneity. The number of participants in the project was greater than in similar studies because other hematological malignancies and autologous HSCT candidates were omitted. Additionally, three separate days were used for the collection of dietary data. As a result, individual variation could more effectively be detected. Despite the strengths, there were also limitations. The cross-sectional design makes the cause-and-effect relationship between the diagnosis of malnutrition and post-transplant outcomes unclear. Considering the differences in ethnicity within other studies and its effect on some parameters for determining malnutrition, such as BMI, FFMI and ASMI, it is necessary to conduct similar studies in different populations in different countries to obtain results with higher clinical significance. While our study aimed to compare the performance of three tools, namely NRS-2002, ESPEN criteria, and GLIM criteria, it is important to acknowledge that these tools serve distinct roles in the nutritional assessment process. NRS-2002 is a recognized screening tool designed to identify patients at risk of malnutrition (13), whereas ESPEN criteria and GLIM criteria are assessments used for diagnosing and stratifying malnutrition (45). We recognize the conceptual challenge in directly comparing screening and assessment tools. However, our study’s scope was tailored to the pre-allo-HSCT context, where early detection of nutritional risk is crucial. As such, these tools were chosen considering their relevance to this specific setting.

## Conclusion

This study demonstrates that the GLIM criterion perfectly agreed with the NRS-2002. The ESPEN criterion had a fair agreement with other two instruments, and it has problems in early diagnosis of malnutrition, especially in the pre-cachexia stage. The agreement of the three tools with each other was also moderate. In the future, it is imperative to undertake rigorous scientific investigations focused on discerning the optimal diagnostic modality for malnutrition and fostering consensus in this regard.

## Data availability statement

The raw data supporting the conclusions of this article will be made available by the authors, without undue reservation.

## Ethics statement

The studies involving humans were approved by Tehran University of Medical Sciences ethics committee. The studies were conducted in accordance with the local legislation and institutional requirements. The participants provided their written informed consent to participate in this study.

## Author contributions

RK and HM designed the study. SM, MB, and AR did data collection. ES did the analysis. HI, SZ-M, and RK wrote the article. SW provided additional scientific analysis and reviewed and edited the manuscript. Finally, HM and SW carefully read the text and tables and made the necessary edits. All authors contributed to the article and approved the submitted version.
